# Supplementary material for: 1,3a,6a-Triazapentalene derivatives as photo-induced cytotoxic small fluorescent dyes
Source: Commun Chem. 2023 Feb 22;6:37. doi: 10.1038/s42004-023-00838-0 (PMC9947109; doi:10.1038/s42004-023-00838-0)
Supplement: Supplementary file 2 — Supplementary Information [file 42004_2023_838_MOESM2_ESM.pdf]

• Supplementary Tables

Supplementary Table 1 | Fluorescence properties of TAP derivatives.

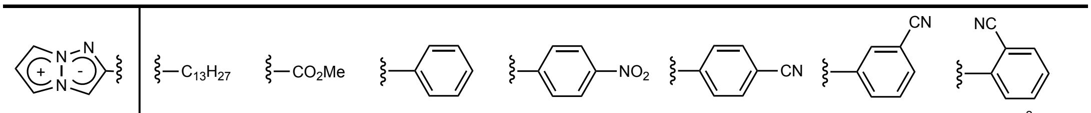

	1a[1]	1b[1]	1c[1]	1d[1]	1e[1]	1f[2]	1g[2]
λ_{abs}^{max}(nm)	N/A	342	326	412	381	327	376
λ_{em}^{max}(nm)	N/A	431	419	556	509	493	515
Φ_F	N/A	0.21	0.030	0.16	0.18	0.24	0.24

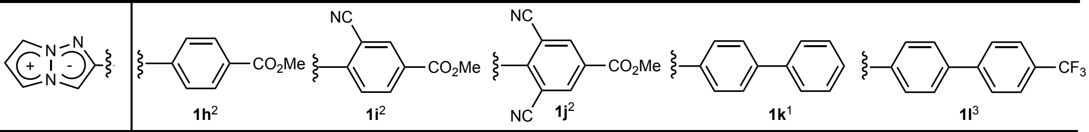

	1h[2]	1i[2]	1j[2]	1k[1]	1l[3]
λ_{abs}^{max}(nm)	376	420	466	345	357
λ_{em}^{max}(nm)	510	572	632	456	506
Φ_F	0.44	0.34	0.096	0.24	0.50

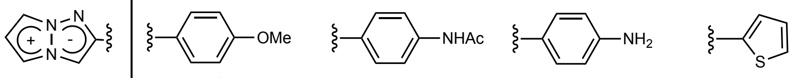

	1m[1]	1n	1o	1p
λ_{abs}^{max}(nm)	330	336	287	341
λ_{em}^{max}(nm)	413	428	429	439
Φ_F	0.062	0.27	0.032	0.057

Florescence properties of TAP derivatives used in Fig. 2 were summarized. The samples used in this study were the same quality as the references. Synthetic procedure, spectral data and charts of these TAP derivatives are available in the supplementary references (see supplementary ref. 1 for **1a-e**, **1k**, and **1m**; see supplementary ref. 2 for **1f-1j**; see supplementary ref. 3 for **1l**). Those of new TAP derivatives **1n-1s** are in this supplementary information for synthetic procedure and compound data, supplementary data 1 for NMR charts, and supplementary data 2 for absorption and fluorescence spectra.

Supplementary Table 2 | Colocalization analysis

Organelle marker	Rcoloc
Calnexin (Endoplasmic reticulum)	0.223
GM130 (Golgi apparatus)	0.315
AIG (Mitochondria)	0.032
LAMP1 (Lysosome)	0.346
Rab5 (Early endosome)	0.350
Rab7 (Late endosome)	0.439
Catalase (Peroxisome)	0.085

The Pearson correlation (Rcoloc) value for organelle markers and **1d** in the colocalization studies were calculated using the colocalization analysis of the ImageJ software.

• Supplementary Figures

Supplementary Figure 1 | UV irradiation

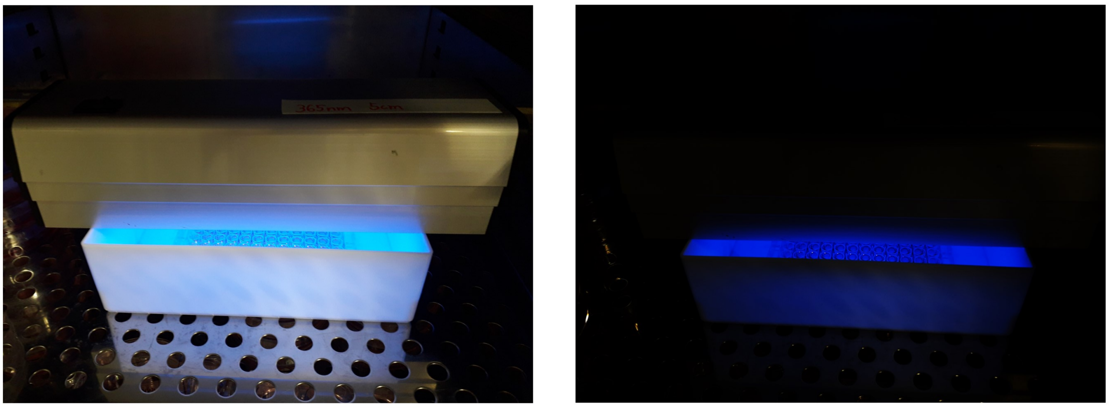

TAP derivatives treated cells were irradiated with UV (Wave length: 365nm, 6 W) light from a distance of 5 cm for 1h in CO_2 incubator.

Supplementary Figure 2 | Cell viability of UV irradiation without compound

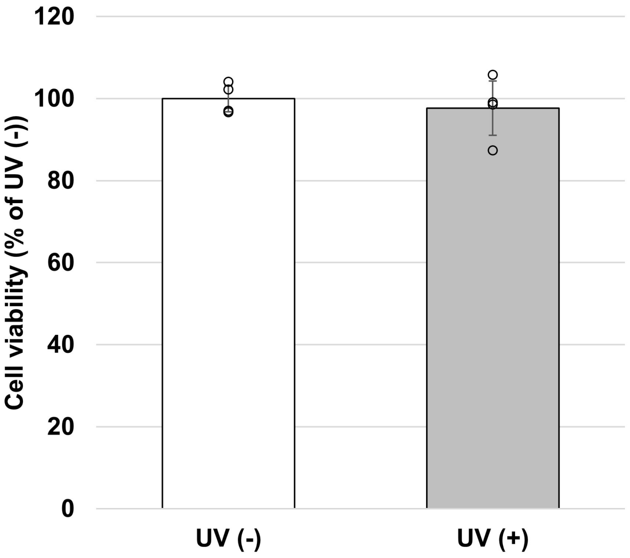

HeLa cells were cultured without compound (Untreated) and cell viability upon UV irradiation was measured by the WST8 assay. N=4 biologically independent samples. The error bars represent the standard deviation of the mean.

Supplementary Figure 3 | Cytotoxicity of UV or Blue LED irradiation with 1d.

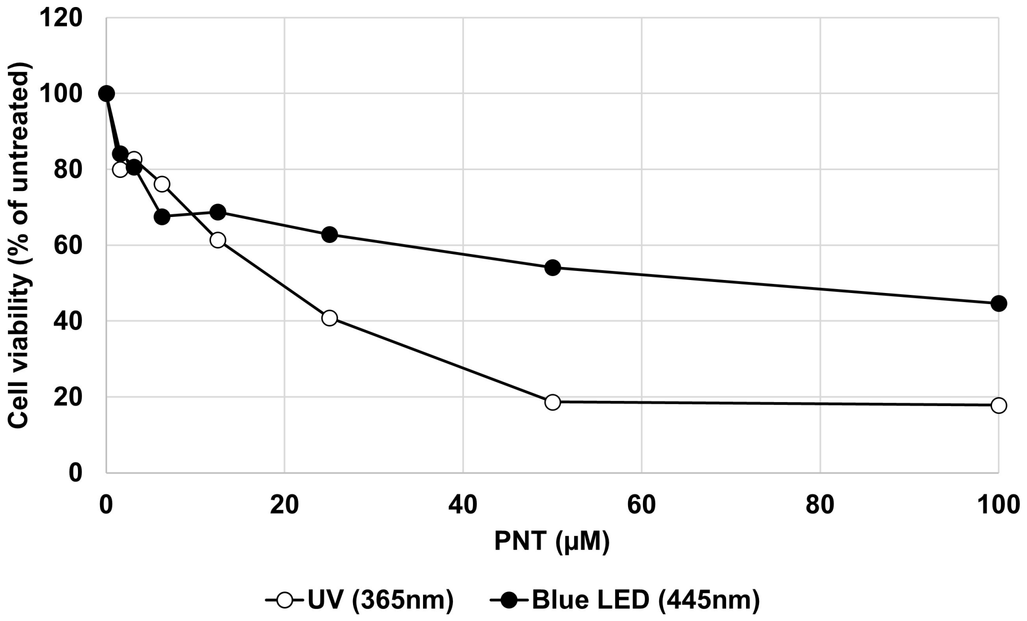

Blue LED irradiation also showed **1d** concentration-dependent photo-induced cytotoxicity, although weaker than UV irradiation.

Supplementary Figure 4 | Dose dependency of photo-induced cytotoxicity under different conditions of 1d treatment

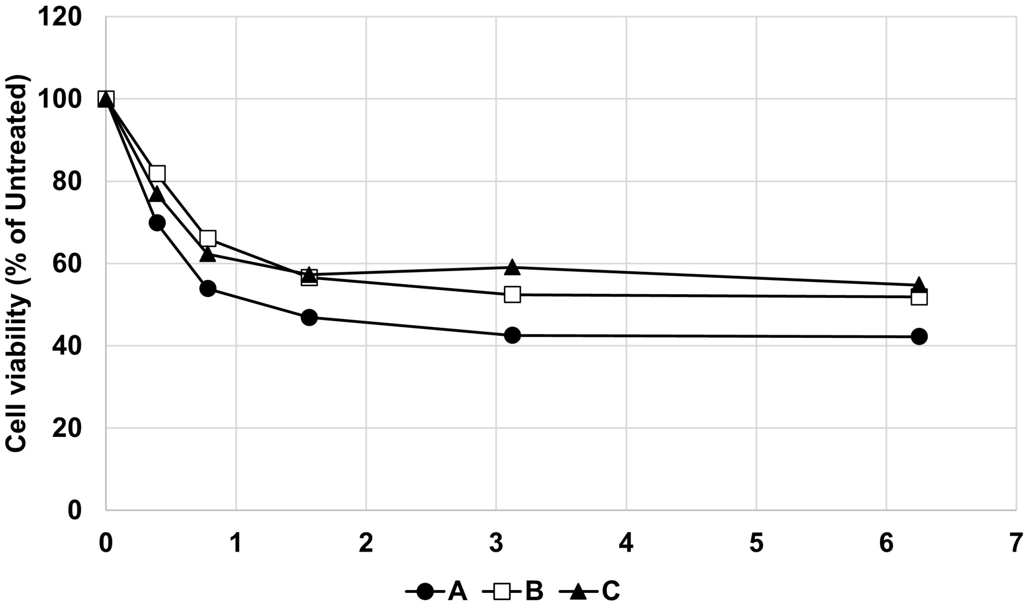

HeLa cells were cultured with several conditions of 1d and the photo-induced cytotoxic effect of UV irradiation was determined by WST8 assay. A: Cells were treated with 1d for 1h, and then UV irradiation 1h as is (total 1d treatment time: 2h), B: Cells were treated with 1d for 1h, and then the medium was replaced before UV irradiation 1h as is (total 1d treatment time: 1h), C: Cells were irradiated UV 1h as soon as 1d added (total 1d treatment time: 1h).

Supplementary Figure 5 | Magnified cell imaging of Fig 4.

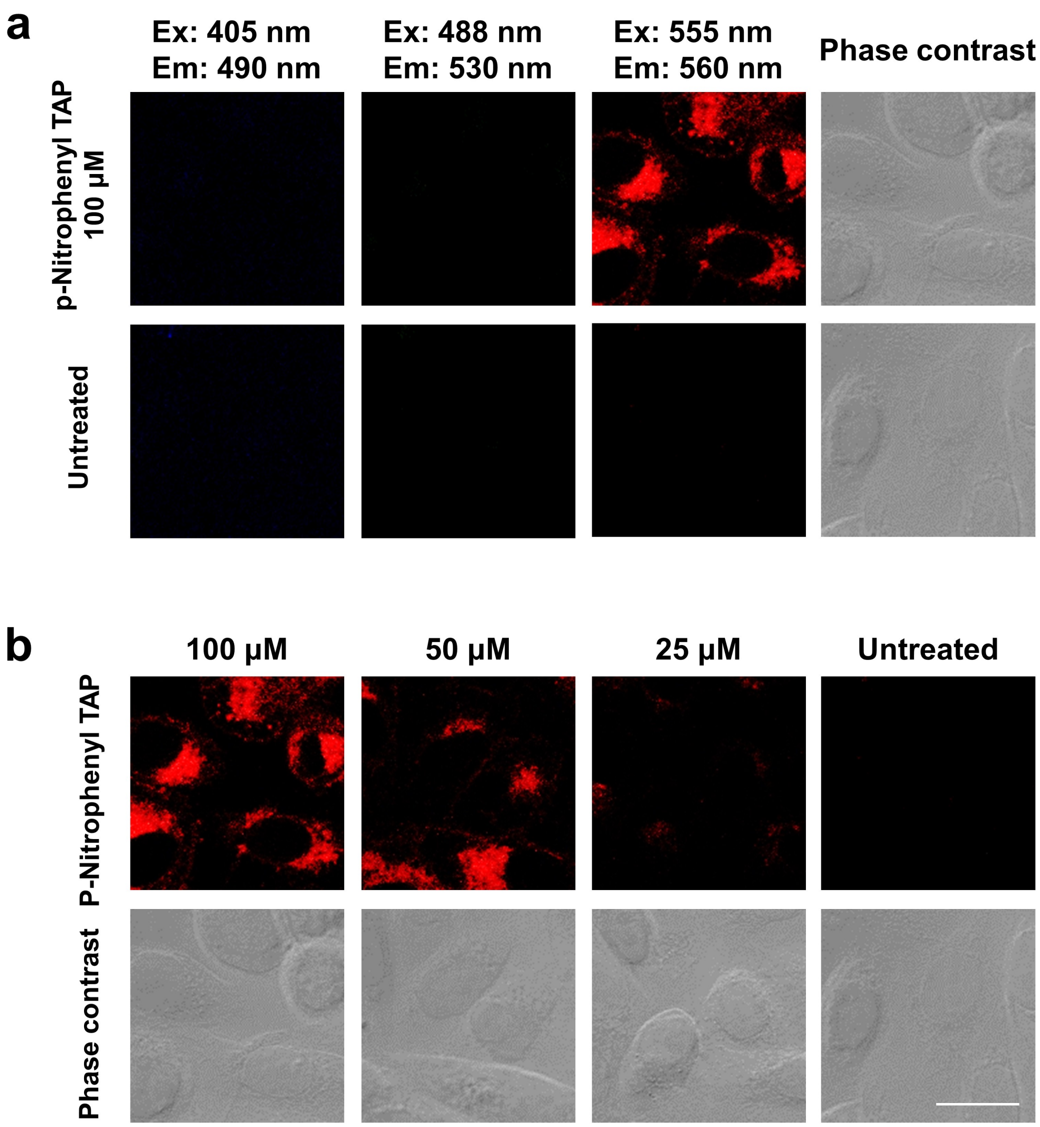

a Magnified cell imaging of Fig 4a in the main text.

b Magnified cell imaging of Fig. 4b in the main text.

Scale bar =20 μm.

Supplementary Figure 6 | Observation of 1d or Alexa555 in HeLa cells.

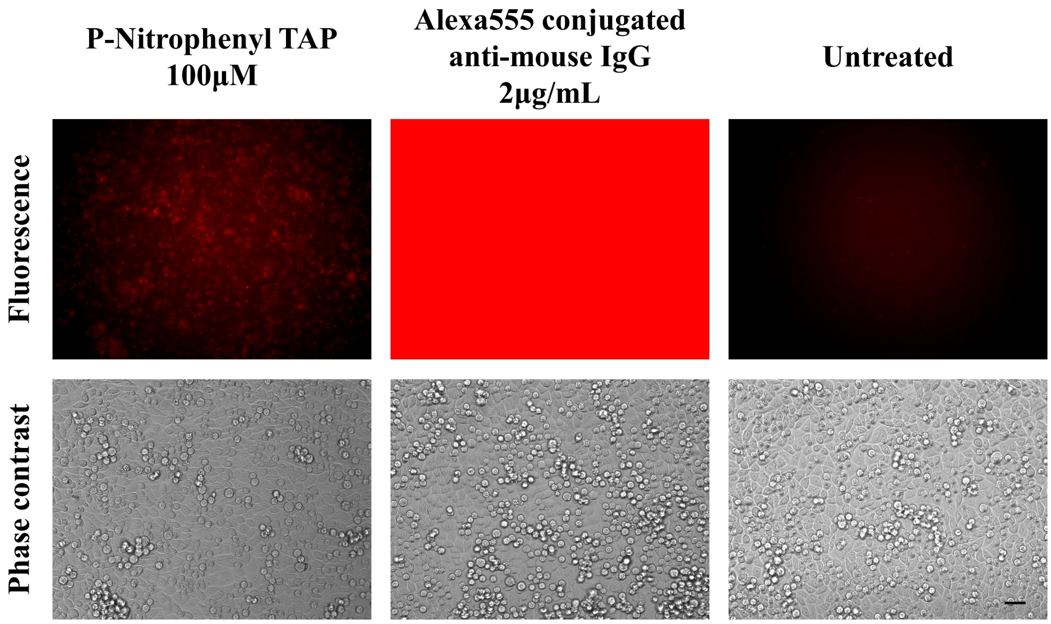

HeLa cells were cultured with 100 μM of 1d or 2μg/mL of Alexa555 conjugated anti-mouse IgG (Cell Signaling Technology) for 1 h, and then the cells were monitored by fluorescent microscopy (BIOREVO BZ-9000; KEYENCE) in fluorescence images with TRITC filter. Scale bar =50 μm.

Supplementary Figure 7 | Localization of 1d in HeLa cells.

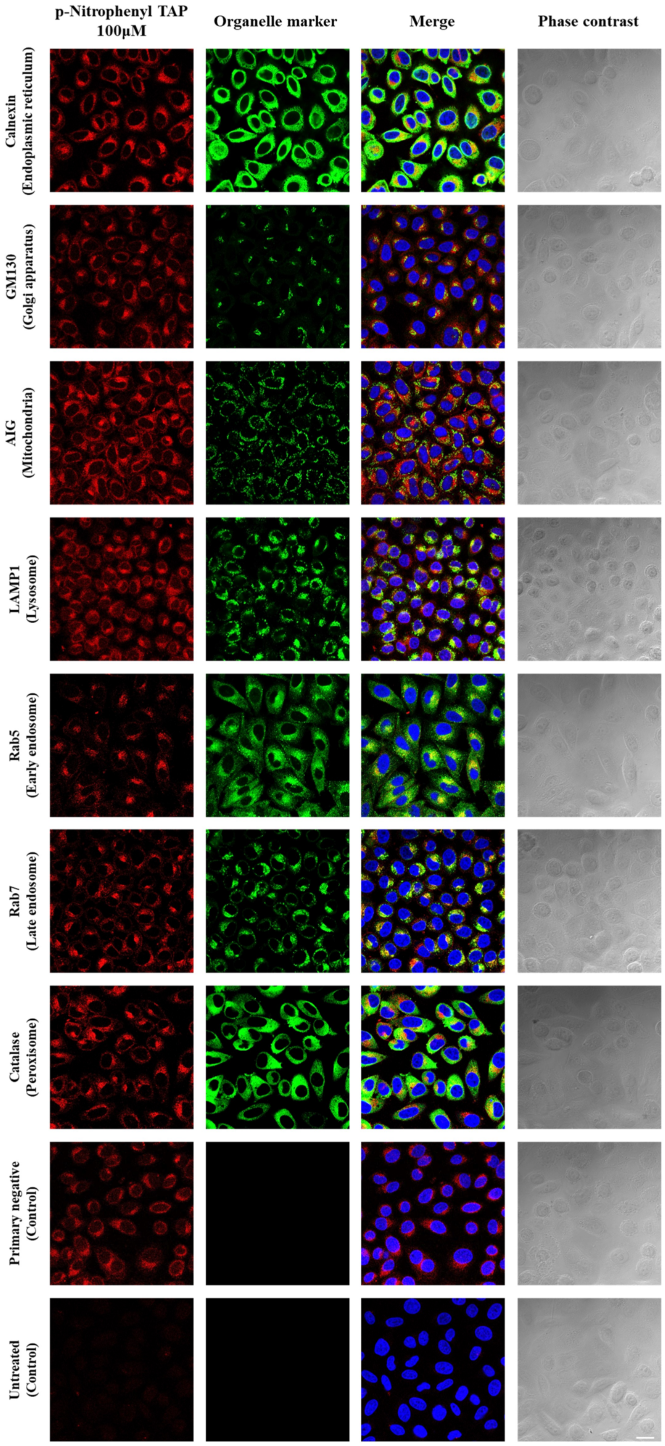

HeLa cells were cultured with 100 µM of **1d** for 1 h, and then the cells were fixed with 4% paraformaldehyde (PFA) after incubation at 4 °C for 24 h. Fixed cells were treated with primary antibodies of organelle markers at 4 °C for 24 h. The cells were washed with phosphate buffered saline

(PBS), and then the cells were incubated with Alexa488 conjugated secondary antibodies and Hoechst33258 for 2h at room temperature. The cells were washed with PBS, and observed using confocal fluorescent microscopy (LSM700, Carl Zeiss). Scale bar =20 µm.

Supplementary Figure 8 | Magnified cell imaging of Fig. 5a

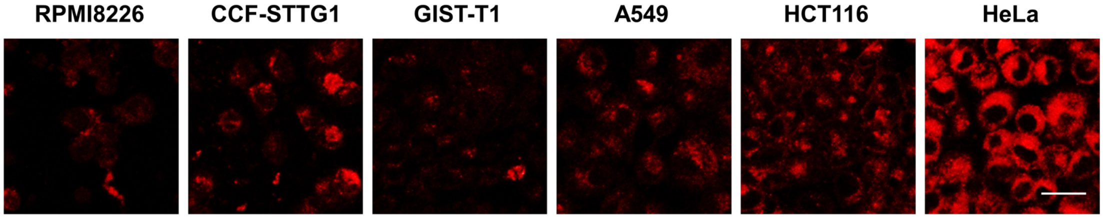

Magnified cell imaging of Fig. 5a in the main text. Scale bar =50 µm.

Supplementary Figure 9 | Production of reactive oxygen species (ROS) in 1d, Rose bengal and Talaporfin after photo-irradiation.

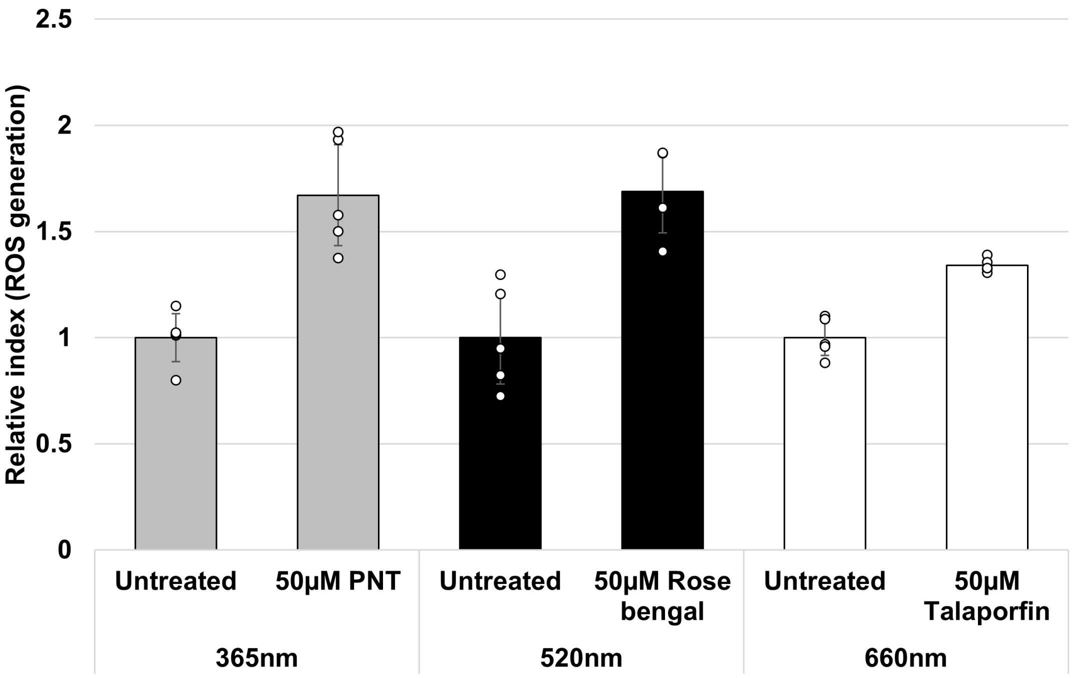

HeLa cells were cultured with 50 µM of compounds for 1 h, and then the cells were iraddiated 365nm (1d) or 520nm (Rose bengal) or 660nm (Talaprfin) for 1h, and then ROS in the cells was measured by a DHE probe (ROS Detection Cell-Based Assay Kit ; Cayman). N=5 biologically independent samples. The error bars represent the standard deviation of the mean.

Supplementary Figure 10 | Magnified cell imaging of Fig7.

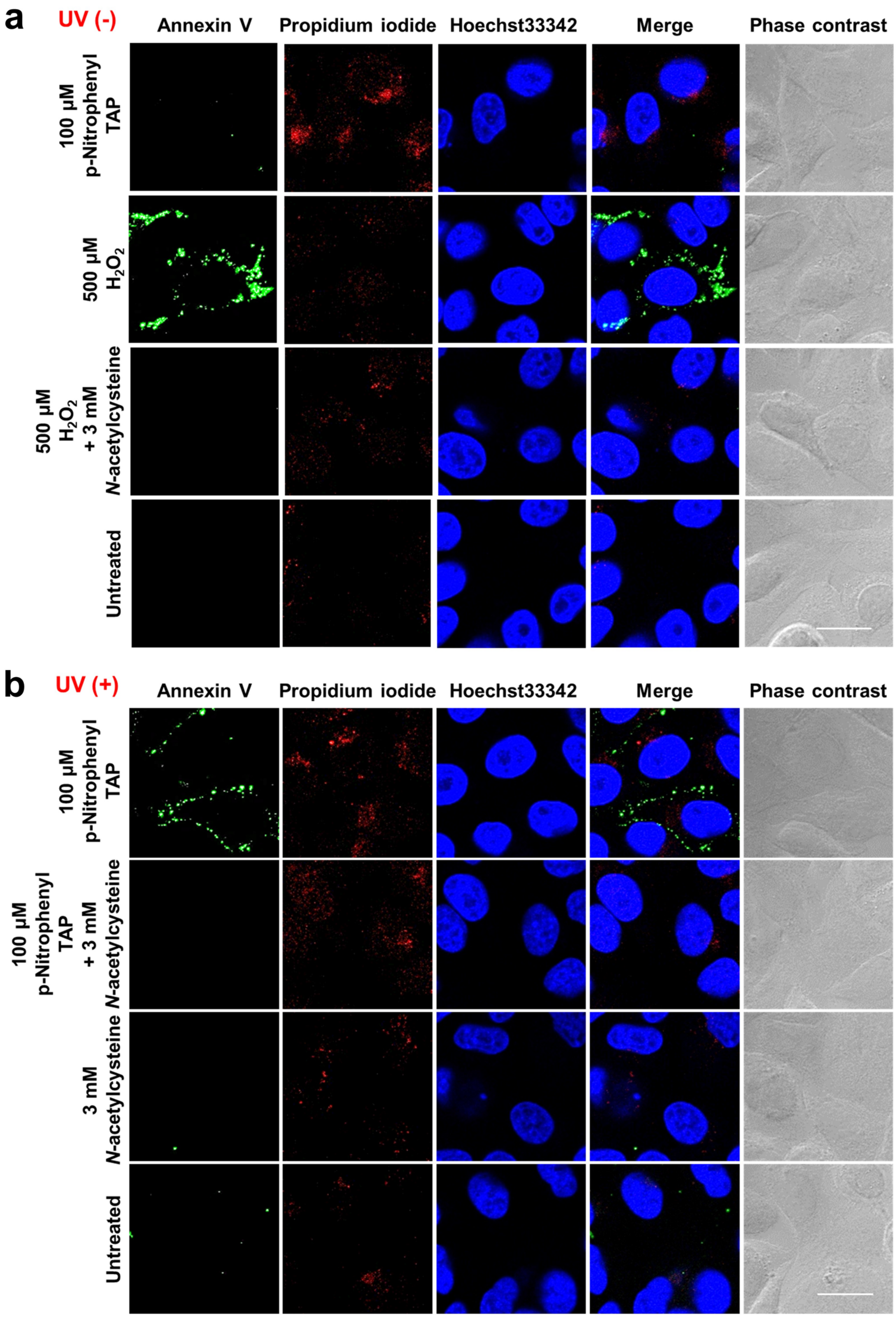

a Magnified cell imaging of Fig. 7a in the main text.

b Magnified cell imaging of Fig. 7b in the main text.

Scale bar =20 μm.

Supplementary Fig. 11 | Magnified cell imaging of Fig. 8c

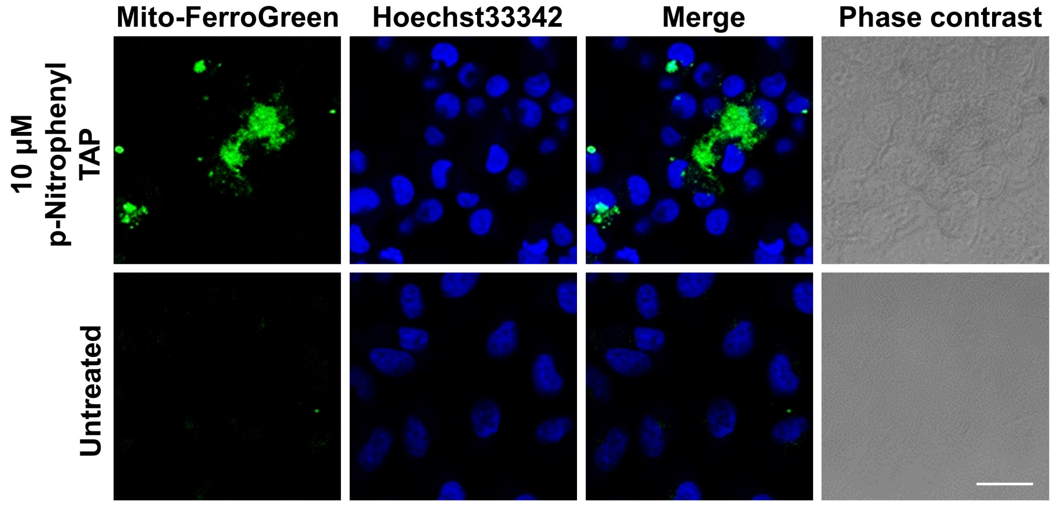

Magnified cell imaging of Fig. 8c in the main text. Scale bar =50 μm.

• Supplementary Methods

General Procedure

All the reactions were carried out in a round-bottomed flask connected to a three-way stopcock or a rubber septum cap under an argon atmosphere. All vessels were evacuated by vacuum pump and then flushed with argon before use. Solutions and solvents were introduced by syringe through a rubber septum. During the reaction, the vessel was kept under a positive pressure of argon. Dry tetrahydrofuran (THF) was freshly prepared by VAC solvent purifier. Infrared (IR) spectra were recorded on JASCO FT/IR-4200 spectrophotometer using KBr plate. Wavelengths of maximum absorbance are quoted in cm^{-1}. ^{1}H NMR spectra were recorded on a Bruker AV-400N (400 MHz) in $CDCl_3$ and C_6D_6. Chemical shifts are reported in part per million (ppm), and signal are expressed as singlet (s), doublet (d), triplet (t), quartet (q), multiplet (m). ^{13}C-NMR spectra were recorded on a Bruker AV–500N (125 MHz) in $CDCl_3$. Chemical shifts are reported in part per million (ppm). For ^{1}H NMR spectra ($CDCl_3$ and C_6D_6), the residual solvent peak was used as the internal reference (7.26 ppm and 7.16 ppm), whereas the central solvent peak was used as the reference (77.16 ppm) for ^{13}C NMR spectra. High resolution mass (HRMS) spectra were recorded on a Thermo Scientific Exactive. All melting points were measured with AS ONE ATM-01. Absorption spectra were recorded on a JASCO V-600 spectrometer and corrected fluorescence spectra were recorded on a JASCO FP-8200 spectrofluorometer. Sample solutions were degassed thoroughly by purging with an Ar gas stream for 30 min prior to the experiments and then sealed in their cells. Fluorescence quantum yields were estimated by using 9,10-diphenylanthracene (9,10-DPA) in cyclohexane (Φ_F = 0.91) as a standard. Analytical thin layer chromatography (TLC) was performed using 0.25 mm E. Merck Silica gel (60F-254) plates. Reaction components were visualized *p*-anisaldehyde in 10% sulfuric acid in ethanol. Kanto Chem. Co. Silica Gel 60N (particle size 0.040–0.050 mm) was used for column chromatography. High-resolution images of cells were obtained using a ZEISS LSM700 (Carl Zeiss) confocal microscope.

Experimental Procedures

General procedure of TAP-forming reaction

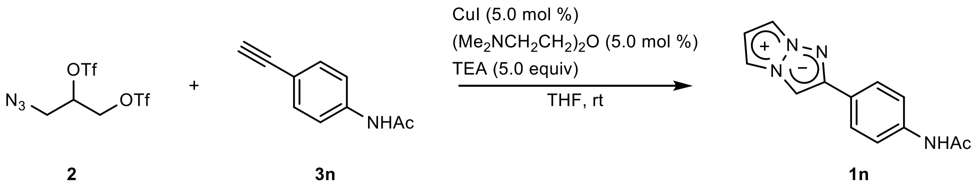

2-*p*-acetylaminophenyl-1,3a,6a-triazapentalene (1n): To a solution of bis[2-(*N*,*N*-dimethylaminoethyl)]ether (19.0 µL, 0.10 mmol) in THF (10 mL) was added copper(I) iodide (19 mg, 0.10 mmol) at room temperature. The mixture was stirred until homogeneous, and 0.62 mL (0.0062 mmol of ligand·copper complex) of resulting mixture was transferred to the flask containing azide **2** (59.0 mg, 0.15 mmol) in THF (12 mL). To the mixture were added TEA (88 µL, 0.63 mmol) and the alkyne **3n** (20 mg, 0.13 mmol) at room temperature, successively. The mixture was stirred for 5 h, diluted with Et_2O, and washed with 10% aqueous solution of NH_3 (x 3). The organic layer was dried over anhydrous $MgSO_4$, filtered, and concentrated under reduced pressure. The residue was purified by flash silica gel column chromatography (hexane/EtOAc = 2/1 to 1/1 containing 1.0% of Et_3N) to give **1n** (11.5 mg, 0.048 mmol, 38%) as a green solid. Mp 105 °C (dec.) (recrystallized from $CHCl_3$); ^1H NMR ($CDCl_3$, 500 MHz): δ 7.72 (d, J = 8.6 Hz, 2H), 7.56 (d, J = 8.6 Hz, 2H), 7.41 (d, *J* = 2.8 Hz, 1H), 7.36 (d, *J* = 0.9 Hz, 1H), 7.30 (br s, 1H), 7.11 (d, *J* = 2.8 Hz, 1H), 6.60 (t, *J* = 2.8 Hz, 1H), 2.20 (s, 3H); ^{13}C NMR ($CDCl_3$, 125 MHz): δ 168.6, 147.3, 138.1, 127.5, 126.6, 120.0, 108.9, 102.4, 101.0, 93.2, 24.8; IR (KBr): 3303, 2102, 1668, 1602, 1537, 1318 cm^{-1}; HRMS (ESI) m/z $[M+Na]^+$ calcd for $[C_{13}H_{12}N_4ONa]^+$ 263.0909, found 263.0905; UV/Vis (CH_2Cl_2): λ_{max} (log ε) = 286 (4.44), 336 (3.68) nm; FL (CH_2Cl_2): λ_{max} = 428 nm. Φ_F = 0.27 (reference to 9,10-DPA; excited at 350 nm).

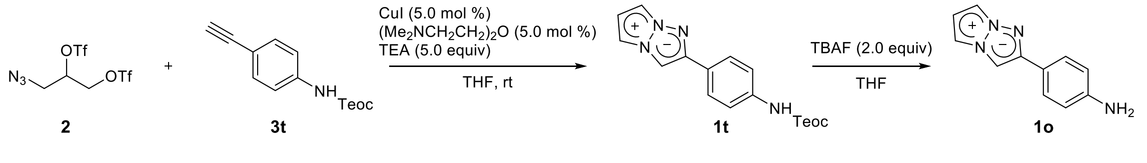

2-*p*-aminophenyl-1,3a,6a-triazapentalene (1o): To a solution of bis[2-(*N*,*N*-dimethylaminoethyl)]ether (19.0 µL, 0.10 mmol) in THF (10 mL) was added copper(I) iodide (19 mg, 0.10 mmol) at room temperature. The mixture was stirred until homogeneous, and 3.8 mL (0.038 mmol of ligand·copper complex) of resulting mixture was transferred to the flask containing azide **2** (420 mg, 1.10 mmol) in THF (38 mL). To the mixture were added TEA (0.52 ml, 3.82 mmol) and alkyne **3t** (200 mg, 0.77 mmol) at room temperature, successively. The mixture was stirred for 6.5 h, diluted with Et_2O, and washed with 10% aqueous solution of NH_3 (x 3). The organic layer was dried over anhydrous $MgSO_4$, filtered, and concentrated under reduced pressure. The residue was purified by

flash silica gel column chromatography (hexane/EtOAc = 9/1 containing 1.0% of Et_3N) to give **1t** (104.9 mg, 0.31 mmol, 45%) as a green solid. To a solution of **1t** (20 mg, 0.05mmol) in THF (0.5 ml) was added TBAF in THF (1.0 M solution, 0.11 ml). The mixture was stirred at room temperature for 21 hours, diluted with Et_2O, and washed with 10% aqueous solution of NH_3 (x 3). The organic layer was dried over anhydrous $MgSO_4$, filtered, and concentrated under reduced pressure. The residue was purified by silica gel column chromatography (hexane/EtOAc = 1/1 containing 1.0% of Et_3N) to give **1o** (4.5 mg, 0.02 mmol, 39%) as a light green solid. Mp 101 °C (dec.) (recrystallized from $CHCl_3$); ^{1}H NMR ($CDCl_3$, 500 MHz): δ 7.56 (dt, J = 8.6, 2.6 Hz, 2H), 7.39 (d, J = 2.9 Hz, 1H), 7.27 (d, J = 1.1 Hz, 1H), 7.07 (d, J = 2.5 Hz, 1H), 6.73 (dt, J = 8.6, 2.6 Hz, 2H), 6.57 (t, J = 2.9 Hz, 1H), 3.77 (br s, 2H); ^{13}C NMR ($CDCl_3$, 125 MHz): δ148.2, 146.8, 127.1, 122.0, 115.3, 108.5, 102.2, 100.6, 92.4; IR (KBr): 3330, 3150, 2359, 1622, 1432, 1290 cm^{-1}; HRMS (ESI): m/z $[M+Na]^+$ calcd for $[C_{11}H_{10}N_4Na]^+$ 221.0803, found 221.0805; UV/Vis (CH_2Cl_2): λ_{max} (log ε) = 287 (4.39) nm; FL (CH_2Cl_2): λ_{max} = 429 nm. Φ_F = 0.035 (reference to 9,10-DPA; excited at 350 nm).

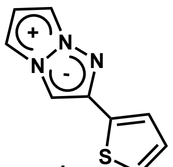

1p

2-(thiophen-2-yl)pyrazolo[1,2-*a*][1,2,3]triazol-8-ium-1-ide (1p)

According to the general procedure, **1p** was obtained from 2-ethynyl-thiophene in 84% yield as a red solid. Mp 71–73 °C (recrystallized from ether); ^{1}H NMR (500 MHz, $CDCl_3$) δ 7.40 (d, J = 2.8 Hz, 1H), 7.36 (d, J = 3.4 Hz, 1H), 7.31 (brs, 1H), 7.30 (d, J = 5.2 Hz, 1H), 7.10–7.04 (m, 2H), 6.58 (t, J = 2.8 Hz, 1H); ^{13}C NMR (125 MHz, $CDCl_3$) δ 142.6, 133.8, 127.4, 125.0, 124.1, 108.7, 102.4, 101.0, 92.9; IR (neat) 3147, 3101, 3069, 1437, 1414, 1381, 1238, 1222, 1034 cm^{-1}; HRMS (ESI) m/z $[M+H]^+$ calcd for $[C_9H_7N_3S+H]^+$ 190.0433, found 190.0435. UV/Vis (CH_2Cl_2): λ_{max} (log ε) = 341 (3.16), 285 (3.97) nm. FL (CH_2Cl_2): λ_{em} = 439 nm; Φ_F = 0.057 (reference to 9,10-DPA; excited at 350 nm).

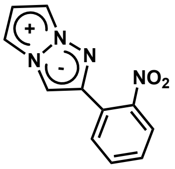

1q

2-*o*-nitrophenyl-1,3a,6a-triazapentalene (1n): According to the general procedure, **1q** (33.7 mg, 73%) was obtained from 1-ethynyl-2-nitrobenzene (35.9 mg, 0.24 mmol) as a dark green solid. Mp 65 °C (dec.) (recrystallized from hexane/EtOAc); ^{1}H NMR ($CDCl_3$, 400 MHz): δ 7.83 (dd, J = 7.8, 1.3 Hz, 1H), 7.72 (dd, J = 8.0, 1.1 Hz, 1H), 7.62 (td, J = 7.7, 1.2 Hz, 1H), 7.50 (td, J = 7.7, 1.2 Hz, 1H), 7.43 (d, J = 2.6 Hz, 1H), 7.31 (d, J = 0.9 Hz, 1H), 7.14 (d, J = 2.9 Hz, 1H), 6.65 (t, J = 2.9 Hz, 1H); ^{13}C NMR ($CDCl_3$, 125 MHz): δ149.5, 142.6, 132.0, 131.3, 129.3, 125.2, 123.7, 109.3, 102.6, 101.3, 95.4; IR (KBr): 3156, 1528, 1430, 1364, 1140, 950 cm^{-1}; HRMS (ESI): m/z $[M+H]^+$ calcd for $[C_{11}H_9N_4O_2]^+$ 229.0726, found 229.0719; UV/Vis (CH_2Cl_2): λ_{max} (log ε) = 283 (4.26) nm. Fluorescence of **1q** was not observed.

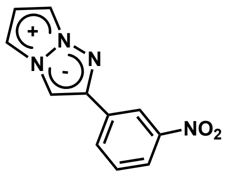

1r

2-*m*-nitrophenyl-1,3a,6a-triazapentalene (1r): According to the general procedure, **1r** (181 mg, 68%) was obtained from 1-ethynyl-3-nitrobenzene (370 mg, 0.97 mmol) as a dark green solid. Mp 117 °C (dec.) (recrystallized from hexane/EtOAc); ^{1}H NMR ($CDCl_3$, 400 MHz): δ 8.59 (t, J = 1.9 Hz, 1H), 8.18 (dd, J = 8.3, 1.3 Hz, 1H), 8.11 (d, J = 7.8 Hz, 1 H), 7.59 (t, J = 8.0 Hz, 1H), 7.51 (s, 1H), 7.47 (d, J = 2.6 Hz, 1H), 7.18 (d, J = 2.8 Hz, 1H), 6.67 (t, J = 2.9 Hz, 1H); ^{13}C NMR ($CDCl_3$, 125 MHz): δ 148.6, 145.3, 133.4, 131.5, 129.7, 122.8, 120.6, 109.5, 102.7, 101.6, 94.0; IR (KBr): 3156, 2345, 1528, 1344, 1253 cm^{-1}; HRMS (ESI) m/z $[M+H]^+$ calcd for $[C_{11}H_9N_4O_2]^+$ 229.0726, found 229.0719; UV/Vis (CH_2Cl_2): λ_{max} (log ε) = 253 (4.19) nm, 319 (3.51) nm; FL (CH_2Cl_2): λ_{max} = 417 nm. Φ_F = 0.0023 (reference to 9,10-DPA; excited at 370 nm).

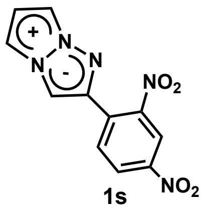

1s

2-*o,p*-dinitrophenyl-1,3a,6a-triazapentalene (1s): According to the general procedure, **1s** (44.5 mg, 31%) was obtained from 1-ethynyl-2,4-dinitrobenzene (100 mg, 0.52 mmol) as a red brown solid. Mp 156-157 °C (dec.) (recrystallized from benzene); ^{1}H NMR (benzene, 400 MHz): δ 7.81 (d, J = 2.1 Hz, 1H), 7.49 (dd, J = 8.7, 2.1 Hz, 1H), 7.46 (d, J = 8.6 Hz, 1H), 6.83 (d, J = 2.6 Hz, 1H), 6.67 (d, J = 1.1 Hz, 1H), 6.15 (d, J = 2.9 Hz, 1H), 5.93 (t, J = 2.9 Hz, 1H); ^{13}C NMR ($CDCl_3$, 125 MHz): δ 147.2, 140.2, 132.0, 130.9, 128.5, 126.2, 119.3, 110.2, 103.2, 102.1, 96.2; IR (KBr): 3113, 2758, 2510, 1523, 1419, 1342 cm^{-1}; HRMS (ESI): m/z $[M+Na]^+$ calcd for $[C_{11}H_7N_5O_4Na]^+$ 296.0396, found 296.0390; UV/Vis (CH_2Cl_2): λ_{max} (log ε) = 281 (4.18) nm; FL (CH_2Cl_2): λ_{max} = 428 nm. Φ_F = 0.040 (reference to 9,10-DPA; excited at 370 nm).

Supplementary Reference

1. Namba, K., Osawa, A., Ishizaka, S., Kitamura, N. & Tanino, K. Direct synthesis of Fluorescent 1,3a,6a-triazapentalene derivatives via click-cyclization-aromatization cascade reaction. *J. Am. Chem. Soc.* **133**, 11466-11469 (2011).
2. Namba, K. *et al.* Synthesis of yellow and red fluorescent 1,3a,6a-triazapentalene and theoretical investigation of optical properties. *Chem. Sci.* **6**, 1083-1093 (2015).
3. Sawada, J. *et al.* Functional 1,3a,6a-triazapentalene scaffold: design of fluorescent probes for kinesin spindle protein (KSP). *Bioorg. Med. Chem. Lett.* **26**, 5765-5769 (2016).
