# Supplementary material for: 1,3a,6a-Triazapentalene derivatives as photo-induced cytotoxic small fluorescent dyes
Source: Commun Chem. 2023 Feb 22;6:37. doi: 10.1038/s42004-023-00838-0 (PMC9947109; doi:10.1038/s42004-023-00838-0)
Supplement: Supplementary file 3 — Description of Additional Supplementary Files [file 42004_2023_838_MOESM3_ESM.pdf]

Description of Additional Supplementary Files

File name: Supplementary Data 1

Description: ^{1}H and ^{13}C NMR spectra of Compound 1n.

File name: Supplementary Data 2

Description: Absorption and Fluorescence emission spectra of 1n.
