# Supplementary figures and images for: 1,3a,6a-Triazapentalene derivatives as photo-induced cytotoxic small fluorescent dyes
Source: Commun Chem. 2023 Feb 22;6:37. doi: 10.1038/s42004-023-00838-0 (PMC9947109; doi:10.1038/s42004-023-00838-0)

## Supplementary Data 1

$^1\text{H}$  and  $^{13}\text{C}$  NMR spectra of Compound 1n-s.

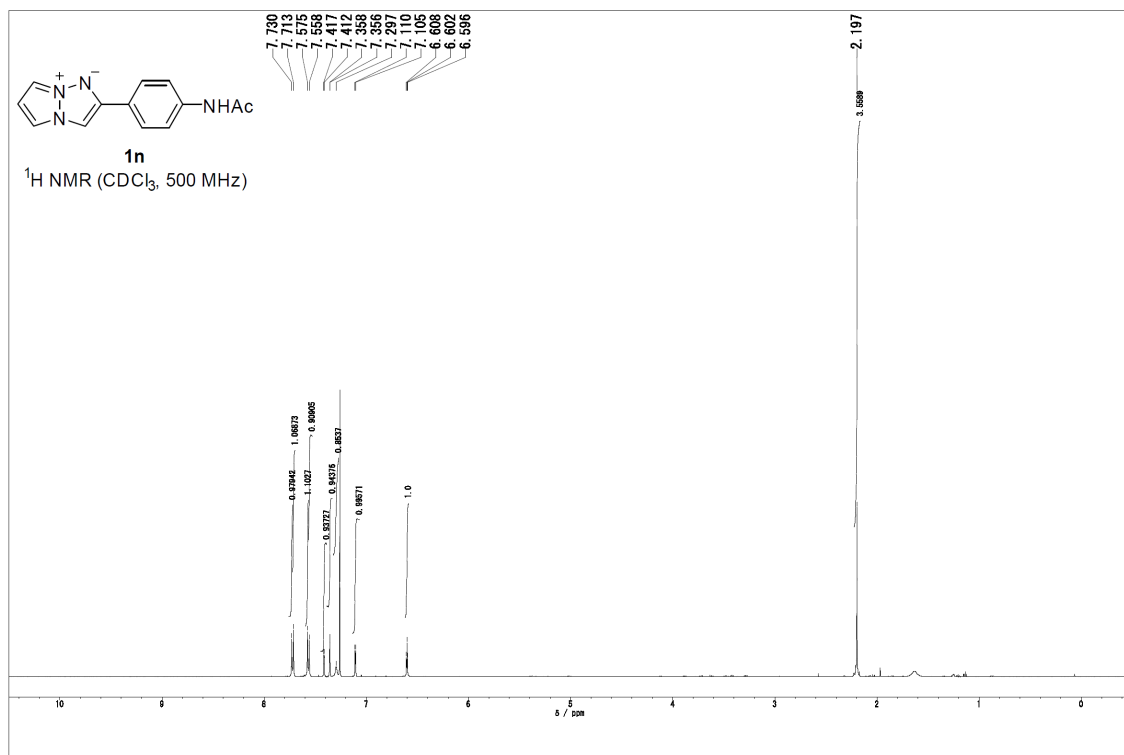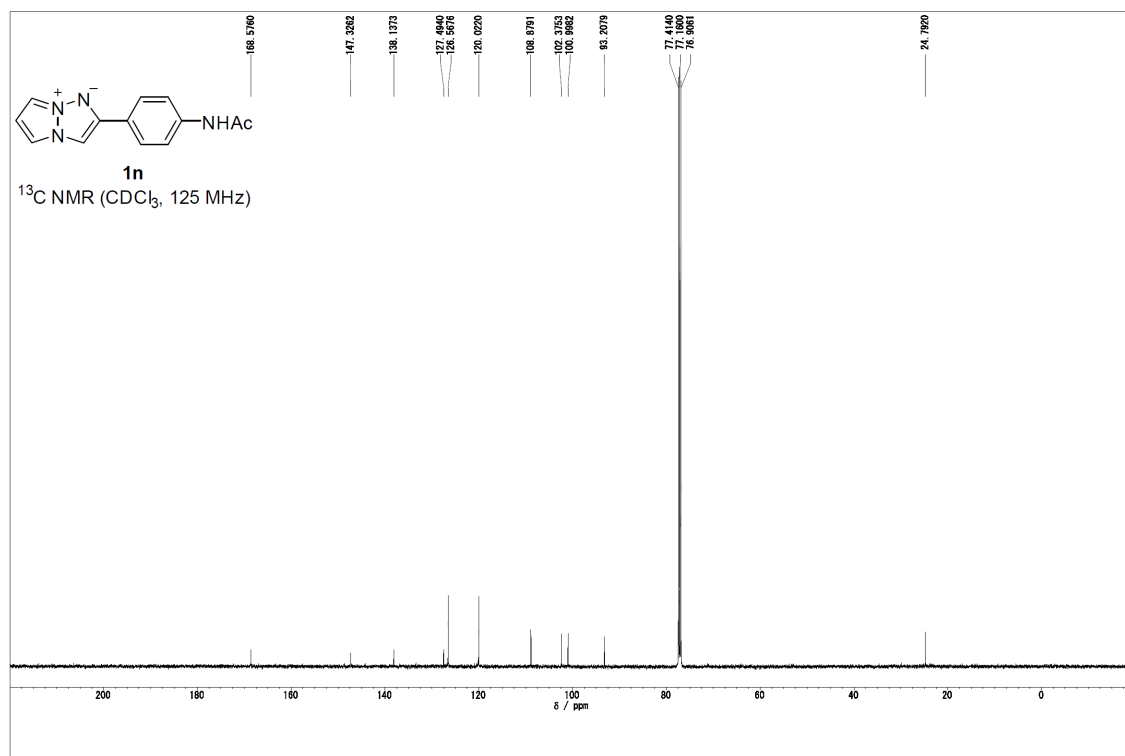



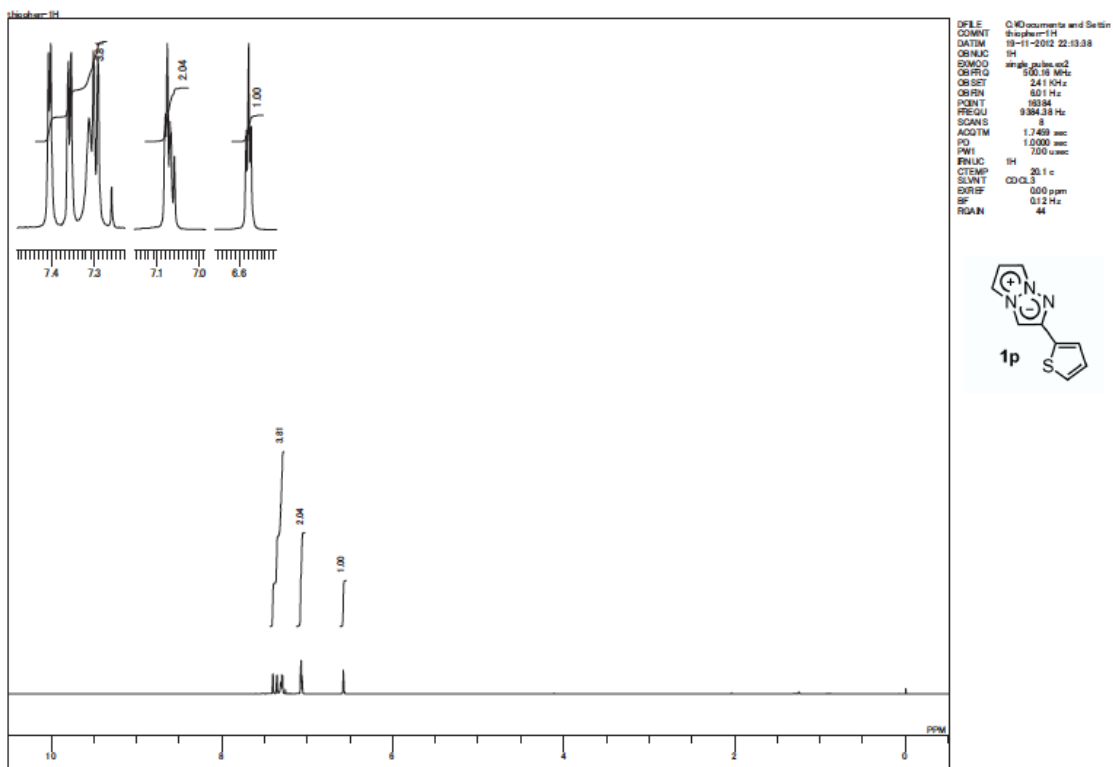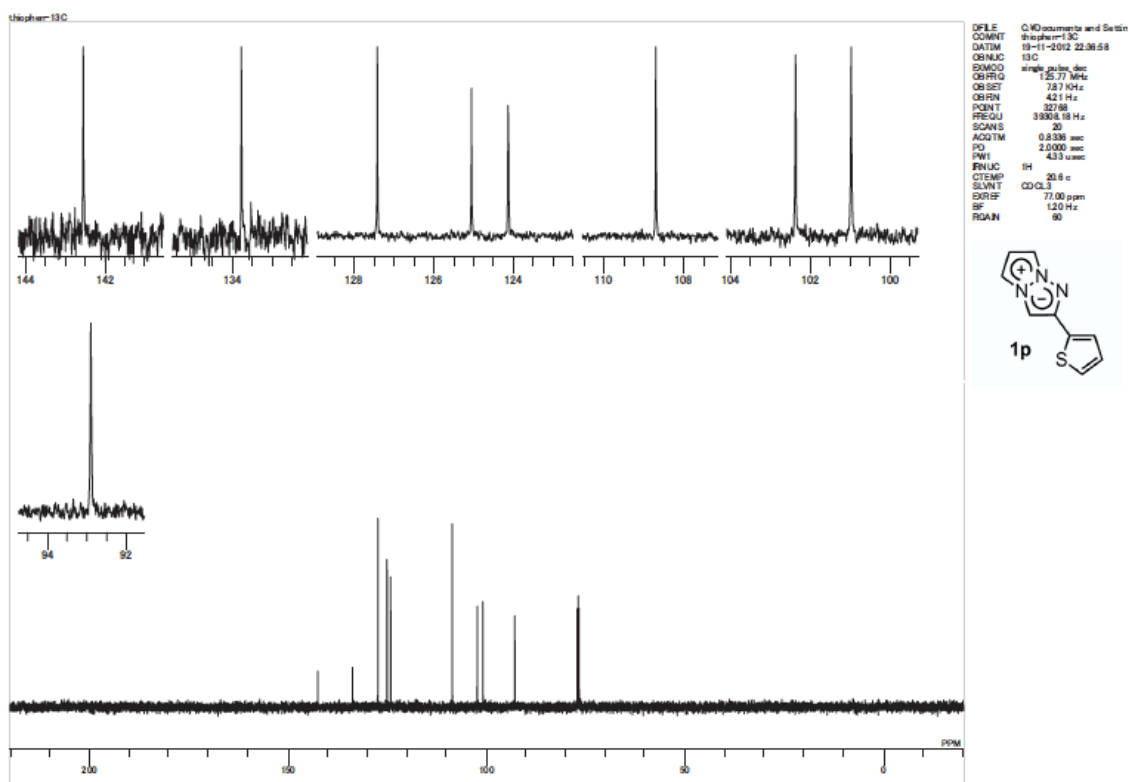

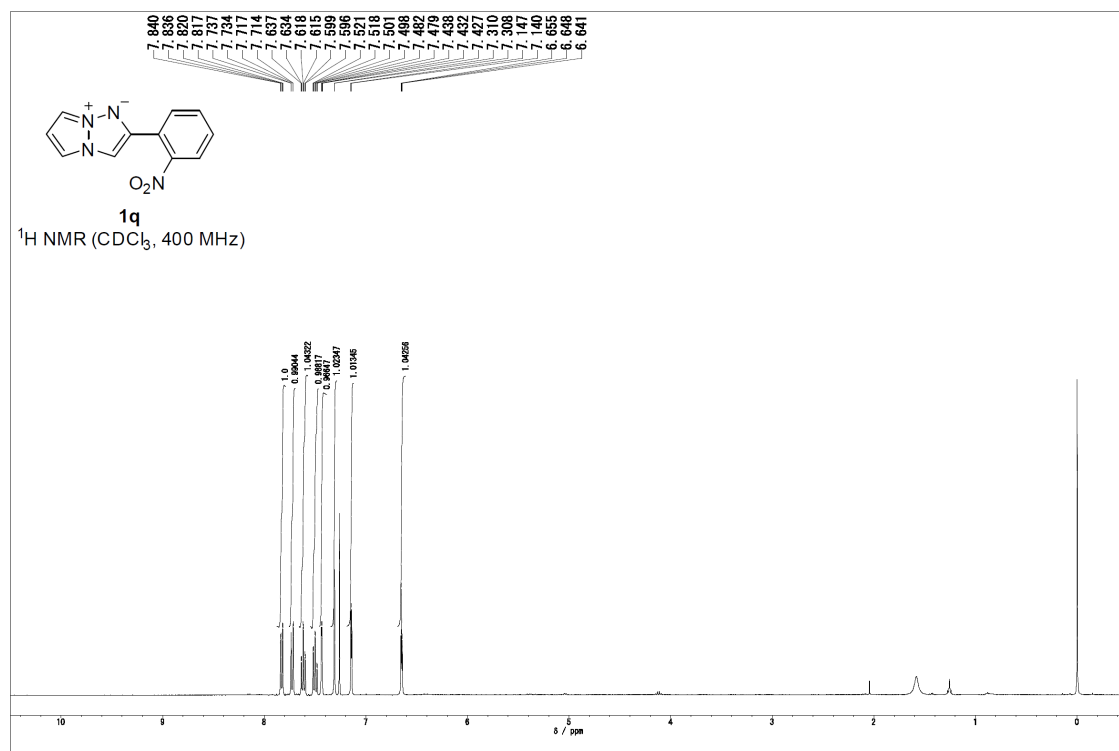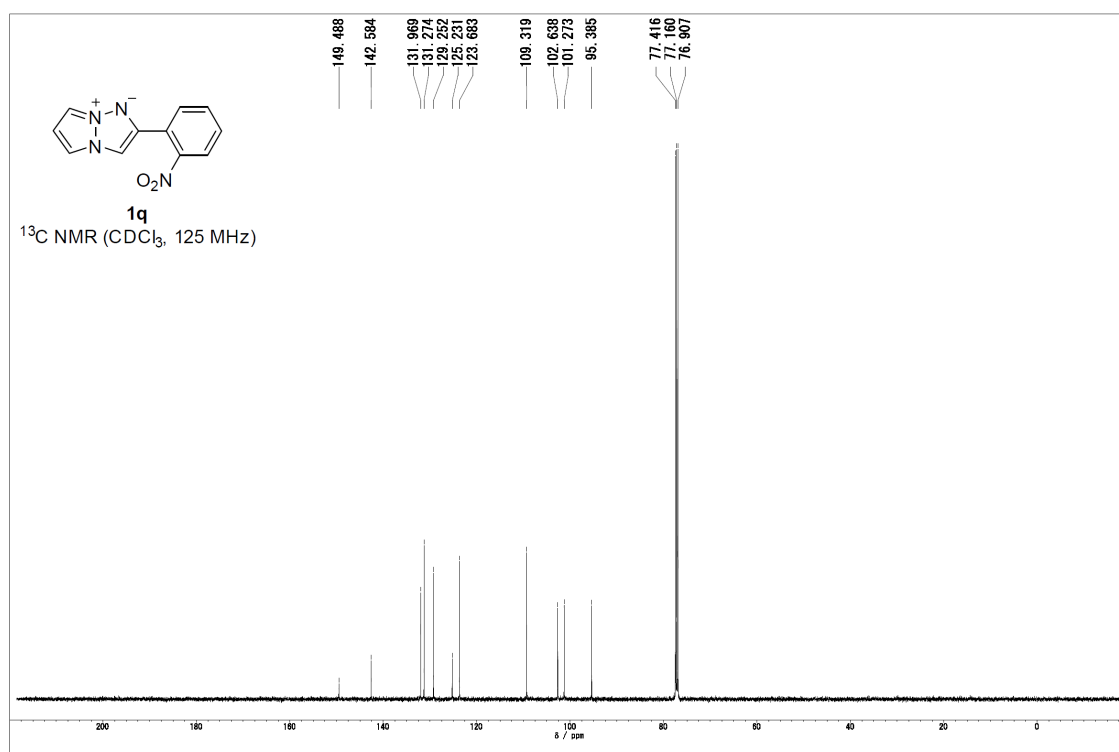

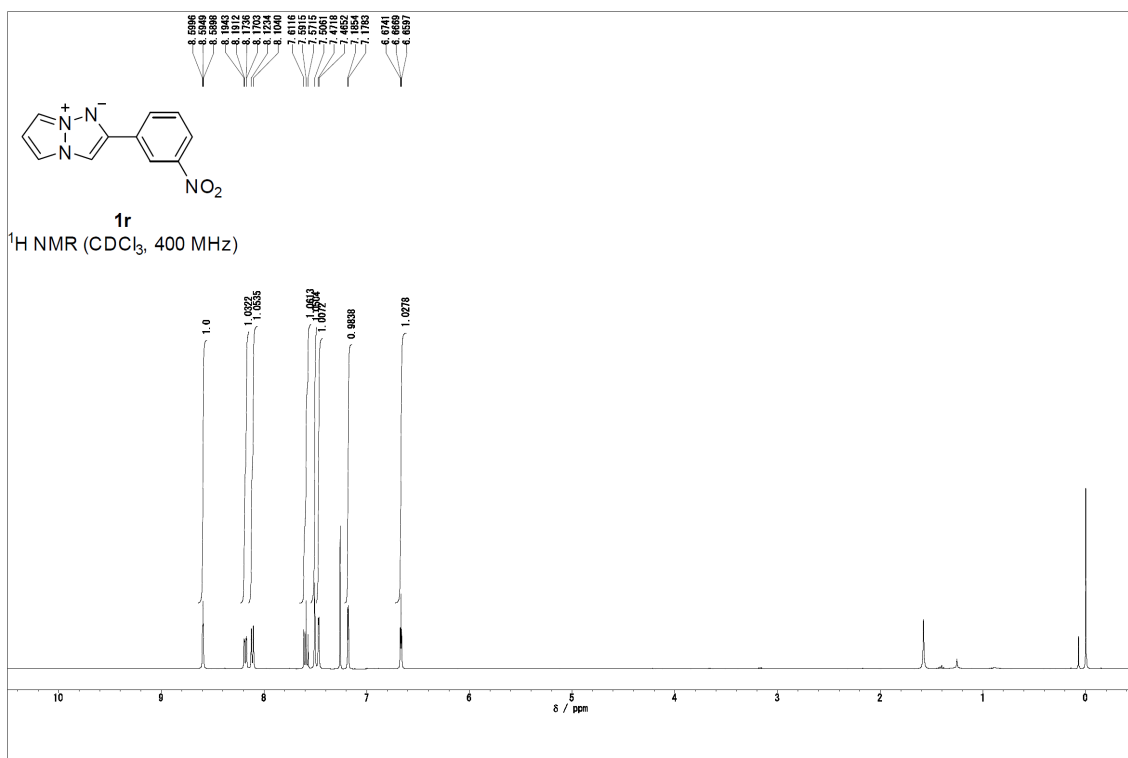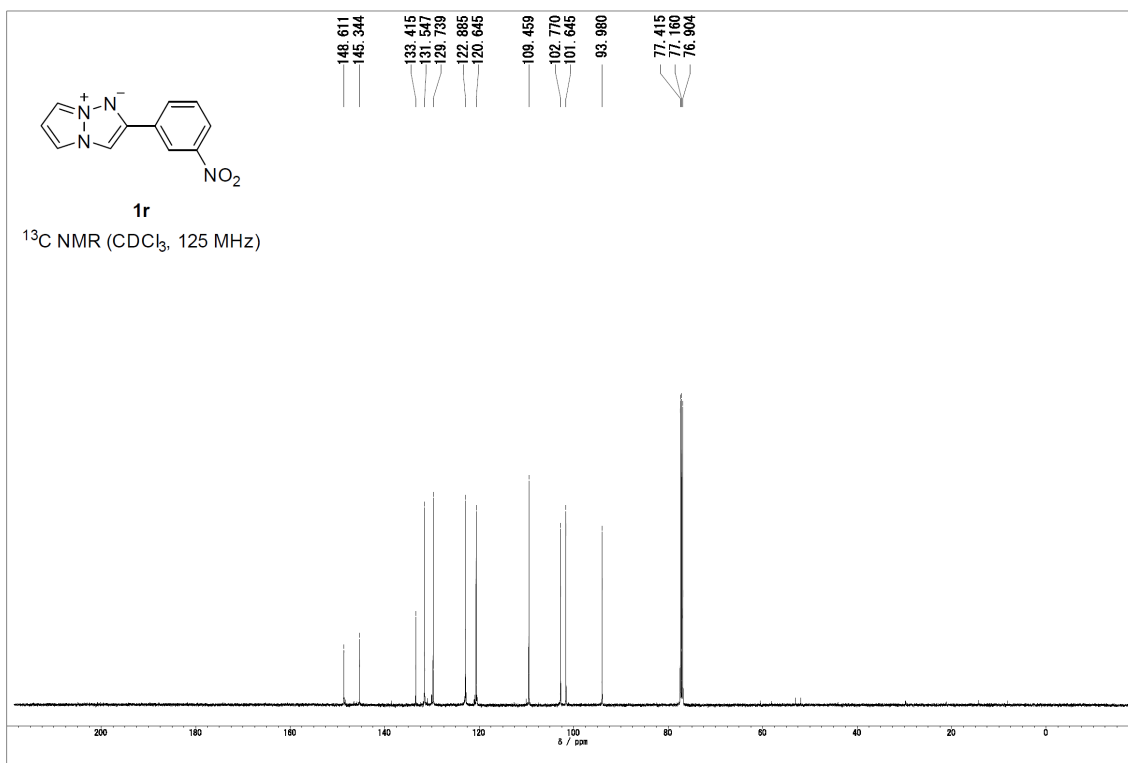

Supplement: Supplementary file 4 — Supplementary Data 1 [file 42004_2023_838_MOESM4_ESM.pdf]
