# Supplementary material for: 1,3a,6a-Triazapentalene derivatives as photo-induced cytotoxic small fluorescent dyes
Source: Commun Chem. 2023 Feb 22;6:37. doi: 10.1038/s42004-023-00838-0 (PMC9947109; doi:10.1038/s42004-023-00838-0)

Supplementary Data 2

Absorption and Fluorescence emission spectra of 1n-s.

Absorption and Fluorescence emission spectra of 1n

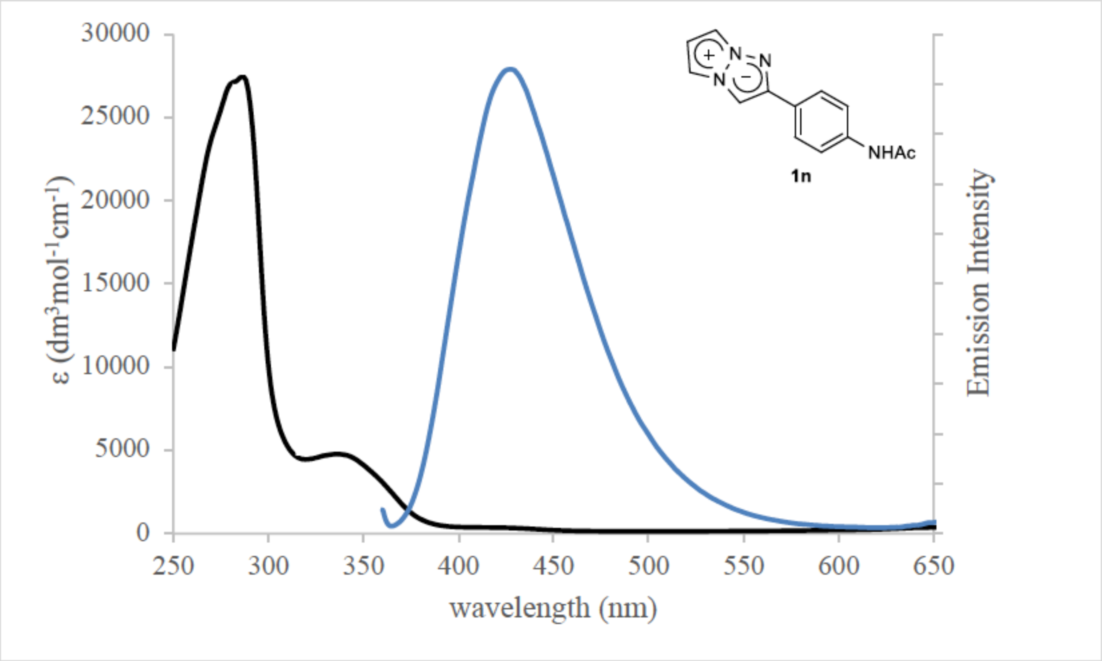

Absorption and Fluorescence emission spectra of 1o.

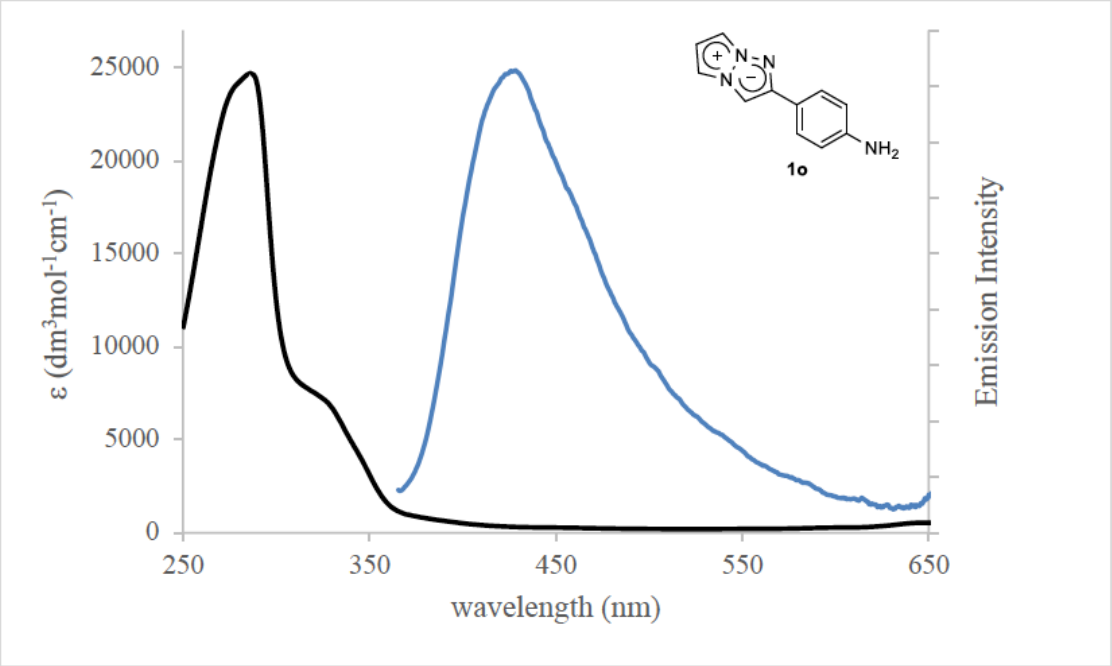

Absorption and Fluorescence emission spectra of 1p.

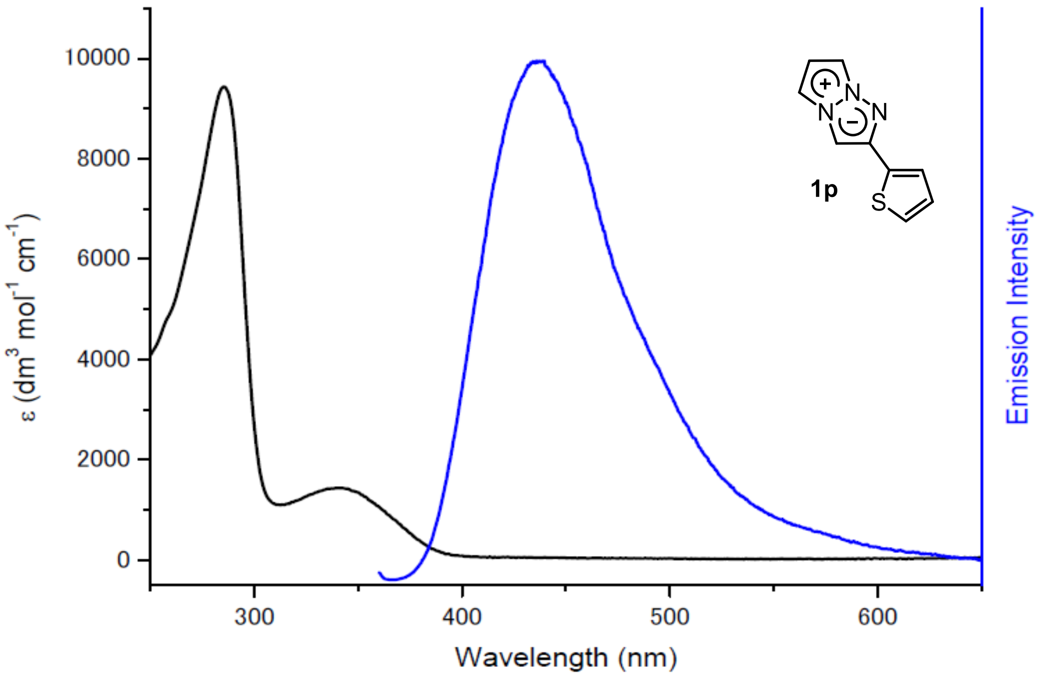

Absorption spectrum of 1q.

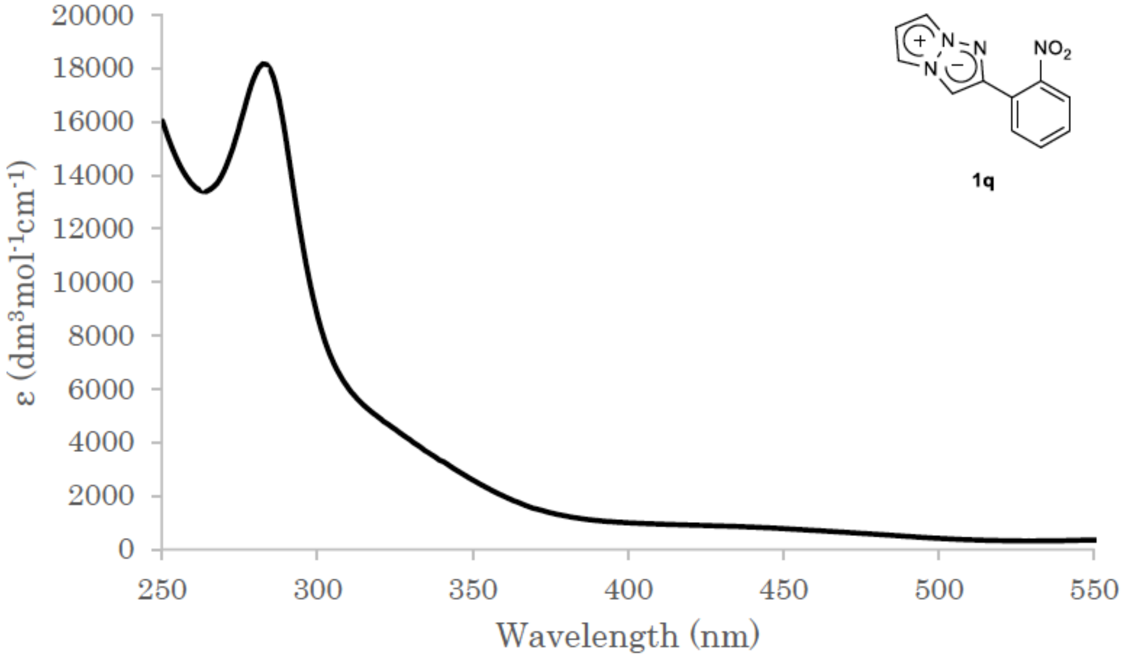

Absorption and Fluorescence emission spectra of 1r.

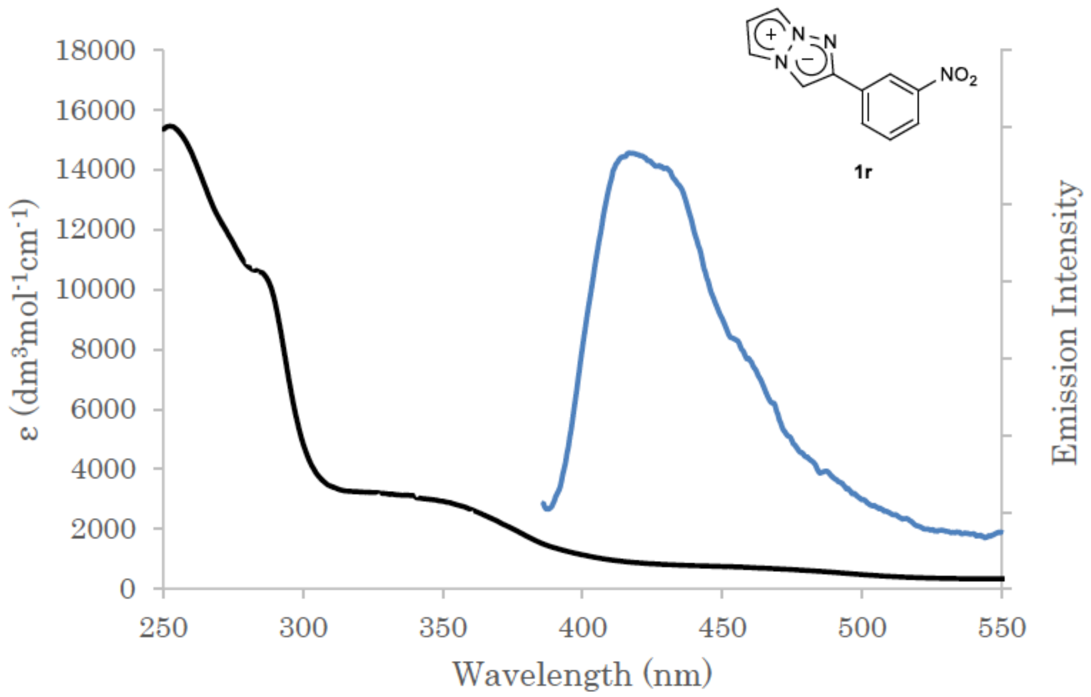

Absorption and Fluorescence emission spectra of 1s.

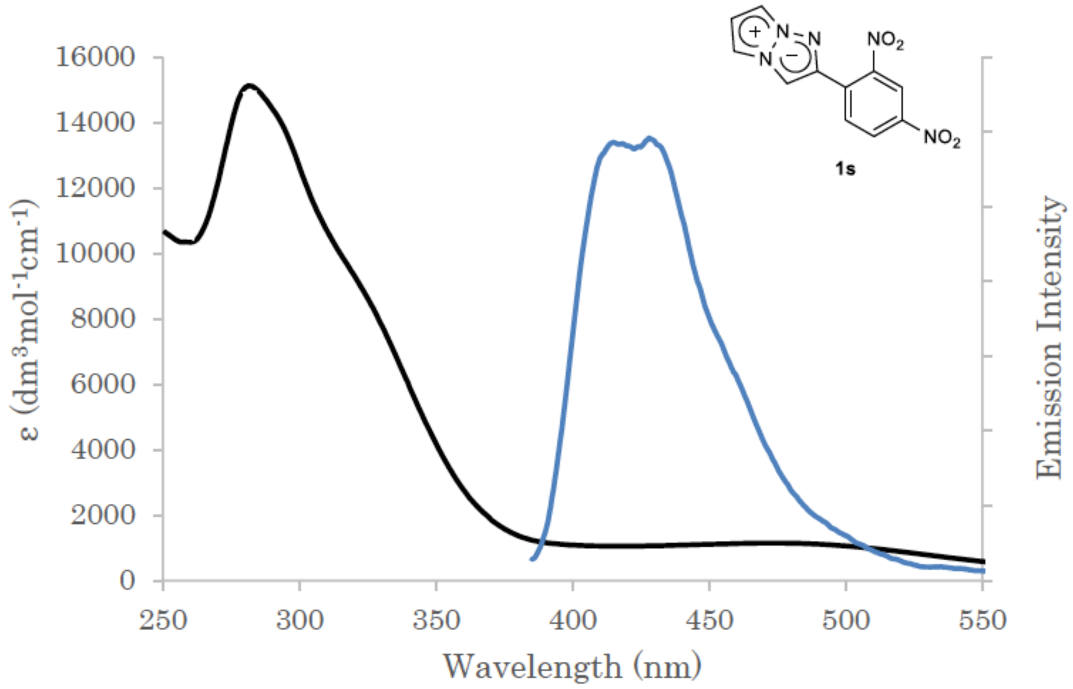

Supplement: Supplementary file 5 — Supplementary Data 2 [file 42004_2023_838_MOESM5_ESM.pdf]
